# Examining cognitive behavioral therapy interventions for unaccompanied minors: a systematic review and qualitative research synthesis

**DOI:** 10.1007/s00787-024-02500-z

**Published:** 2024-06-27

**Authors:** Dafne Morroni, Pinelopi Konstantinou, Chrysilia Gkleka, Angelos P. Kassianos, Maria Karekla

**Affiliations:** 1 Department of Psychology, University of Cyprus, PO Box 20537, Nicosia, 1678 Cyprus; 2 Department of Psychology, School of Sciences, University of Central Lancashire (UCLan), Larnaca, Cyprus; 3 Department of Nursing, Cyprus University of Technology, Limassol, Cyprus

**Keywords:** Unaccompanied minors, Quality of life, Acceptability, Third wave, Systematic review

## Abstract

**Background:**

This systematic review examined the evidence on effectiveness and acceptability of cognitive behavioral therapy (CBT) interventions in improving quality of life (QoL) and psychological well-being of unaccompanied minors (UM).

**Methods:**

PubMed, Scopus, Embase, ProQuest, PsycInfo, PsycArticles, and Open Dissertations databases were used to identify quantitative and qualitative studies. The Effective Public Health Practice Project (EPHPP) and Critical Appraisal Skills Programme (CASP) tools were used for quality assessment. Narrative synthesis and qualitative research synthesis were carried out to collate the findings.

**Results:**

18 studies were included. Two studies examined QoL, and five studies examined acceptability of interventions. Most quantitative studies (*n* = 10) were appraised as methodologically weak. Trauma-Focused CBT appears to have the most evidence demonstrating effectiveness in ameliorating symptoms of post-traumatic stress disorder, depression, and anxiety. Promising findings (i.e., increased mindfulness and psychological flexibility) were observed for third wave interventions but further replication is required.

**Conclusions:**

The literature is tainted by under-powered studies, lacking blinding, and follow-up assessments. Female UM remain largely underrepresented. This review calls for a drastic augmentation of high quality quantitative and qualitative research focusing on augmenting QoL and examining acceptability rather than merely aiming for psychological symptom reduction in UM to enhance overall well-being and functionality. The research protocol was registered in PROSPERO (registration number: CRD42021293881).

**Supplementary Information:**

The online version contains supplementary material available at 10.1007/s00787-024-02500-z.

## Introduction

By the end of 2022, 51,700 unaccompanied minors (UM) were forcibly displaced worldwide, an 89% increase from the previous year [1]. UM are children and adolescents under the age of 18 who have been uprooted from their homes against their will and separated from both parents [2]. UM flee from their homes due to various threats such as forced marriages, recruitment as child soldiers, war, direct or indirect exposure to violence, an unstable political climate, or persecution [3]. What sets UM apart from other migrants and accompanied refugee minors is that they must cope with the loss of positive reinforcers in their lives accompanied by harsh stressors on many levels (personal, family, social, societal etc.). UM are forced to cope with these stressors usually at a very young age and without parental or caretaker support [4]. Furthermore, UM experience a major transition of fleeing their home during a critical period of physical and mental development whilst having to survive and adjust to new contexts [5]. Consequentially, UM are more vulnerable, and it is no surprise that they display more psychopathology than refugee children who are accompanied by at least one parent [6]. Substantial research on prevalence of mental health (MH) difficulties in this population suggests that more than 80% of UM, are frequently diagnosed with post-traumatic stress disorder (PTSD), followed by depression and anxiety disorders [7, 8], traumatic grief and general behavioural problems (i.e., violent or aggressive behavior, breaking rules, fighting; [9]. Oftentimes, UM present with an amalgamation of symptoms without meeting a specific diagnosis [10]. Other than evidence-based guidelines from the National Institute for Health and Care Excellence (NICE) [11–13], there are no official guidelines from established organisations on the treatment of MH difficulties of UM. NICE guidelines recommend Cognitive Behavior Therapy (CBT) for mild to severe depression and Trauma-Focused CBT (TF-CBT) for PTSD in youth. CBT [14] and TF-CBT [15] are well-established evidence-based therapeutic approaches in the treatment of children and adolescents dealing with PTSD [16], depression [17], and anxiety [18]. CBT currently consists of three “waves” or generations. “Second wave” CBT combines cognitive therapy with behavior therapy aiming to help individuals learn how to identify and change maladaptive thinking patterns that negatively impact their behavior and emotions [19]. “Third wave” CBT refers to a new generation of CBT approaches that focus on the overall advancement of psychological and behavioral processes involved in well-being instead of the dissipation of psychological symptoms [20]. Third wave CBT interventions include: Acceptance and Commitment Therapy (ACT; [21, 22]), Dialectical Behavior Therapy (DBT; [23, 24]), Functional Analytic Psychotherapy (FAP; [25, 26]), Compassion-Focused Therapy (CFT; [27, 28]), and Mindfulness-Based Cognitive Therapy (MBCT; [29]). Recent research suggests promising results for the use of third wave therapies with young people [30, 31].

Despite promising interventions tackling MH difficulties (i.e., PTSD, depression, anxiety), there is relatively little research consideration for investigating other indices of MH such as quality of life (QoL) in UM which may be a driving factor contributing further to mental health symptom presentation [32]. QoL is a multifaceted concept which constitutes a person’s appraisal of their somatic well-being, emotional state, degree of autonomy, quality of social relationships, private belief systems, and connection with their surroundings [33]. According to Akinyemi et al. [34], QoL along with occupational status, were the largest threats to the MH of refugees. Researchers have advocated for a need to move away from MH symptom reduction emphasis and pathologizing, and instead focus on improving QoL, well-being, and functionality [34, 35]. This shift towards QoL rather than a syndromal approach, is of importance as it is line with recent advancements in the field of psychology. Researchers are calling for an ‘idionomic future of cognitive behavior therapy’ [36] and the use of evolutionary models consisting of various dimensions (i.e., affect, cognition, attention, self, motivation, overt behavior) and levels (i.e., biophysiological, sociocultural) whilst also taking context into account [36, 37].

As well as enhancing QoL, well-being, and functionality, it is of critical importance to assess the acceptability of psychological treatments in refugee populations, particularly due to the stigma surrounding mental illness [38, 39]. Understanding acceptability of an intervention is crucial as it can inform clinicians about factors that promote participation and potential barriers to treatment delivery [40]. Acceptability is a multifaceted construct which can be examined using several methods, based on the theoretical construct in which it is being evaluated [41]. In healthcare, treatment acceptability includes a person’s level of commitment towards an intervention (e.g., adherence, dropout), as well as how a person perceived and experienced an intervention [42, 43]. To evaluate treatment acceptability, qualitative methodology has been used as it can elucidate participant beliefs regarding a treatment which may help to define the components of a treatment that are regarded as meaningful and to appraise cultural sensitivity [44].

To summate, the gaps in the literature thus far are three-fold. Firstly, although the effectiveness of CBT interventions has been investigated [45], research has focused on the abatement of psychopathological symptoms rather than the improvement of QoL in UM. Secondly, despite empirical and theoretical evidence that they could be beneficial for UM, third wave therapies have not been explicitly investigated. Thirdly, as previous reviews merely aggregated qualitative findings related to acceptability of interventions [45], this review will go a step further by carrying out a rigorous qualitative research synthesis (QRS) with the aspiration to develop new insights.

### The present study

The systematic review primary objectives are two-fold: (1) To examine whether CBT and third wave interventions improve the QoL for UM, and (2) To evaluate whether CBT and third wave interventions are acceptable for UM. In addition, to be consistent and comparable with previous research, a secondary objective is to examine the evidence regarding the effectiveness of CBT and third wave interventions in improving psychopathology symptoms (i.e., PTSD, depression, anxiety) for UM.

## Method

The guidelines of Preferred Reporting Items for Systematic Reviews and Meta-Analyses (PRISMA) were followed for the review processes.

### Eligibility criteria

The PICO model [46, 47] was used as the search strategy tool for this systematic review. Population (P): The maximum age for participants was 20 years old, either asylum seekers or refugees, and arrived unaccompanied in the host country. No restrictions were placed on the country of origin of participants. Intervention (I): Studies delivering CBT-based interventions, such as: TF-CBT, Teaching Recovery Techniques (TRT), and third wave CBT interventions (i.e., ACT, DBT, CFT, MBCT) individually or in group therapy formats. Narrative Exposure Therapy studies were excluded as they were not considered CBT-based [48]. Comparison (C): Within-subjects (i.e., no control) or between-subjects comparisons (i.e., CBT-based studies compared to controls or other therapies). Outcomes (O): For the first objective, primary outcomes included changes in measures of QoL from pre- to post-intervention and follow up time-points. Primary outcomes for objective two included qualitative evaluation of acceptability of interventions by UM, therapists, and other stakeholders. Primary outcomes for secondary objective included changes in PTSD, depression, and anxiety measures, from pre- to post-intervention and follow-up time points.

Case studies, case series, randomized controlled trials (RCT), controlled clinical trials (CCT), and uncontrolled pre-post studies were eligible for selection. Both published and unpublished (e.g., dissertations) quantitative and qualitative studies were included in the review. Exclusion criteria consisted of conference abstracts and presentations. Language of articles included were English, Greek, Italian, and French as these constituted languages that the first author or author team speak at intermediate or advanced levels.

### Search strategy, study selection, and synthesis

Relevant studies were identified by searching the following databases: PubMed, Scopus, Embase, ProQuest, PsycInfo, PsycArticles, and Open Dissertations (EBSCO). Google Scholar was also searched for any un-identified studies. Clinical trials were also searched using the ClinicalTrials.gov website. No restriction was set on their publication date. Searches were conducted until February 2024. Search terms based on the title and abstract included (see Online Resource 1): unaccompanied, refugees, minors, adolescent, child, youth, teenager, cognitive behavioral, psychotherapy, therapy, treatment, intervention, counselling, CBT, asylum seeker, displaced, migrants, immigrants, emigration, third wave.

Articles were screened for eligibility by the first author (DM) at all screening stages (i.e., title screening, abstract screening, and full-text screening). At all stages, an additional author (PK) screened 20% of the studies, independently. Inter-rater reliability (IRR) was calculated using the percent agreement and Cohen’s kappa [49]. A substantial agreement was observed between the two screeners in title screening (*IRR* = 90%; *k* = 0.95), an almost perfect agreement in abstract screening (*IRR* = 95%; *k* = 0.89) and moderate agreement in full-text (*IRR* = 87%; *k* = 0.58). Any discrepancies were resolved in research team consensus meetings.

For quantitative data, a narrative synthesis [50, 51] was used to provide descriptive information, summarize, and explain findings of included studies, and collate the extracted evidence. For qualitative data, the QRS approach was chosen as the most appropriate method to develop new insights and interpretations [52]. The QRS includes four stages: description, analysis, synthesis, and interpretation. Initially, the first author (DM) compared characteristics and main findings of studies using a descriptive summary and table. Next, line by line coding of the data was carried out independently by two authors (DM, CG) to record initial thoughts. Data included both participant quotes as well as findings in narrative prose written by researchers. For the analysis phase, the codes were grouped together to develop first order themes which presented key aspects across studies. Following this, the synthesis phase involves combining themes across studies to create a new perspective. Thus, first-order themes were drawn together into composite second order themes. These were constructed through a repetitive process of moving between the raw data, codes, and themes to examine similarities and differences across studies. A matrix was used to record first and second order themes and their prevalence across studies. At the final interpretation stage, themes were compared and condensed to generate a third order theme (overarching concept). Here, the researchers sought to move away from aggregation, towards a novel interpretative understanding of the underlying processes and mechanisms involved in the effectiveness and acceptability of CBT and third wave interventions for UM. This was achieved through a process of careful consideration and reframing of underlying ideas and concepts, alongside revisiting original data.

Across all stages of the analytic process, the authors (DM, CG) were mindful to stay true to the voices of participants included in the synthesis. The final thematic structure was developed by DM, supported by continual discussion, reflection, and checking with CG. The enhancing transparency in reporting the synthesis of qualitative research (ENTREQ) statement was used to enhance transparency in reporting of the QRS [53].

### Data extraction and quality assessment

Microsoft Excel was used to extract and chart the data (see Online Resource 2). The quality assessment of all included studies was conducted by two authors (DM, CG). IRR evaluating consistency among raters yielded good agreement for quantitative studies (*IRR* = 94%) and substantial agreement for qualitative studies (*IRR* = 80%). Discrepancies were discussed in research team meetings to reach consensus. For the QRS, a second author coded 10% of the transcripts to assess intercoder agreement [54]. Having a strong inter-coder (ICR) agreement is important for the trustworthiness of the study findings [55]. Krippendorff’s alpha was calculated as it can be used to include more than two coders and nominal data [56]. In addition, Krippendorff’s alpha was selected as it can assess reliability in cases where multiple codes were applied to a single data segment [57]. Based on recommendations from O’Connor & Joffe (2020) [57], the Statistical Package for Social Science V29.0 (SPSS; Inc., Chicago, IL) was used to calculate the ICR. However, the agreement observed between the two coders was not considered satisfactory (*a* = 0.03). Quantitative studies were appraised using the Effective Public Health Practice Project Quality Assessment Tool for Quantitative Studies (EPHPP; [58]) The EPHPP tool is comprised of eight segments of rating: selection bias, study design, confounders, blinding, data collection methods, withdrawals and dropouts, intervention integrity, and analyses. Qualitative studies were appraised using the Critical Appraisal Skills Programme Qualitative Research Checklist (CASP; [59]). The CASP checklist appraises the applicability, reliability, and validity of published qualitative research based on ten questions focusing on the aim of the research, methodology, research design, recruitment strategy, data collection, relationships between researcher and participants, ethical considerations, data analysis, findings, and the value of the research. The quality of studies using mixed methods were appraised using both tools, respective to their quantitative and qualitative components.

## Results

### Study characteristics

**Fig. 1:**
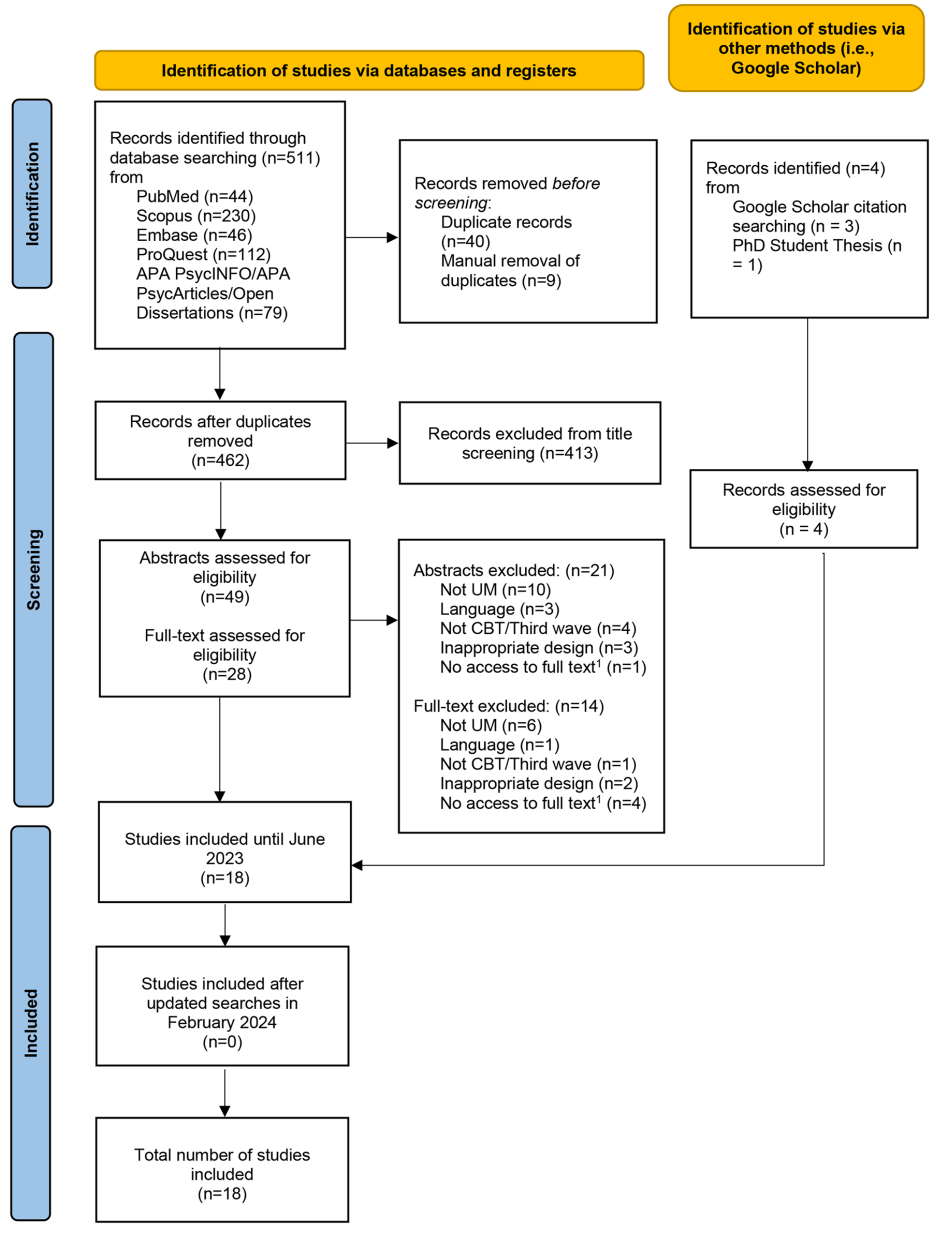
PRISMA flow diagram Note.^1^ An attempt was made to retrieve the full-text by conducting the corresponding author of the article but failed

Searches identified a total of 511 records. After the removal 483 records (462 duplicates, 21 abstracts excluded), the full texts of 28 studies were assessed for full-text screening with 18 finally included (See Fig. 1). Studies included varied in methodology adopted: most studies were quantitative (*n* = 12, 66.7%), followed by mixed methods (*n* = 5, 27.8%), and qualitative (*n* = 1, 5.6%). Two studies were published in 2005 (*n* = 2, 11.1%) and the rest were published between 2015 and 2023 (*n* = 16, 88.9%) All studies were carried out in developed countries with most being conducted in Germany (*n* = 6, 33.3%). Almost half of the included studies implemented a cohort study design (*n* = 8, 44.4%) and most were carried out in community or outpatient settings (*n* = 11, 61.1%). Most studies included UM that originated from different countries (*n* = 15, 83.3%). Sample sizes ranged from one participant to 147 (*Mean* = 34.6). Although most studies included female UM in their sample (*n* = 14, 77.8%), from a total of 670 participants, females were greatly underrepresented (*n* = 142, 21.2%). The age of participants ranged from 4 to 20 years old (*Mean* = 16.22, *SD* = 1.29).

The type of intervention predominantly used was TF-CBT (*n* = 10, 55.6%), with group therapy being the most common treatment modality (*n* = 11, 61.1%). For group therapy studies, the duration of intervention ranged from 4 to 35 weeks (*Mean* = 9.2 weeks) with each group lasting between 60 and 120 min. Studies of individual sessions intervention ranged from 1 to 48 sessions (*Mean* = 12.2 sessions), with sessions lasting between 60 and 100 min. From the included studies, less than half had follow-up assessments ranging from one to six months (*n* = 7, 38.9%). With regards to inclusion and exclusion criteria, some studies required participants to have PTSD symptoms to be included (*n* = 10, 55.6%), a PTSD diagnosis (*n* = 4, 22.2%), or were excluded if they presented with severe mental illness such as psychosis or acute suicidality (*n* = 7, 38.9%). With regards to language, either interpreters were used (*n* = 6, 33.3%), intercultural mediators (*n* = 3, 16.7%), bilingual therapists/research assistants (*n* = 2, 11.1%), or UM were excluded due to not speaking the language of host country (*n* = 5, 27.8%). The characteristics of included studies can be found in Table 1.

**Table 1:** Characteristics of included studies

Study	Country	Study Design	Intervention	Population	Setting	Results	Limitations
Ehntholt et al., 2005	United Kingdom	Case-control	CBT Group 6 sessions 60 min	*n* = 26 (6 μm) Age = 11–15 Mixed (17 male, 9 female) Kosovo, Sierra Leone, Turkey (Kurdish), Afghanistan, Somalia	School	Significant decrease in PTSD symptoms. Significant reduction in intrusive symptoms. Significant decrease in behavioral difficulties.	Small sample size. Questionnaires used not validated with this population. Loss of participants before follow-up assessment.
King & Said, 2019	United Kingdom	Cohort	Third wave Group 35 weeks	*n* = 14 (all UM) Age = 14–17 Mixed (1 female) Afghanistan, Ethiopia, Sudan, Somalia	Child and adolescent MH service	Only four UM showed reliable improvement on questionnaire.	Small sample size. No follow-up assessment.
Mongelli, 2019	Italy	RCT	ACT Group 7 weeks 90 min	*n* = 12 (all UM) Age = 13–18 All male Mali, Gambia, Egypt, Bangladesh, Ivory Coast, Venezuela	Accomodation units	Significant reduction in PTSD and depression symptoms. Significant increase in psychological flexibility and mindfulness.	Small sample size. Questionnaires used not validated with this population. No follow-up assessment.
Patel et al., 2022	USA	Cohort	TF-CBT Individual 1–48 sessions 60–90 min	*n* = 122 (all UM) Age = 4–19 Mixed (76 female) Central America (79%)	Government facilities and children’s centre	Significant reduction in trauma symptoms and emotional difficulties.	No follow-up assessment. High drop-out rate. No statistical comparison with control or active comparator.
Pfeiffer & Goldbeck, 2017	Germany	Cohort	TF-CBT Group 6 weeks 90 min	*n* = 29 (all UM) Age = 14–18 All male Afghanistan	Child welfare programme	Significant reduction in PTSD symptoms	Small sample size. No follow-up assessment. No statistical comparison with control or active comparator.
Pfeiffer et al., 2018	Germany	RCT	TF-CBT Group 6 weeks 90 min	*n* = 99 (all UM) Age = 14–18 Mixed (43 male, 3 female) Afghanistan, Eritrea, Gambia, Pakistan, Albania, Syria, Somalia, Sudan, Iraq, Nigeria, Ghana	Child and adolescent welfare agencies	Significant reduction in PTSD and depression symtoms	Small number of female UM. No follow-up assessment. Intervention fidelity should have been audio or video taped.
Pfeiffer et al., 2019	Germany	RCT	TF-CBT Group 6 weeks 90 min	*n* = 99 (all UM) Age = 14–19 Mixed (43 male, 3 female) Afghanistan, Syria, Gambia, Somalia, Iran, Ethiopia, Pakistan, Angola, Nigeria, Ivory Coast, Ghana, Guinea, Guinea-Bissau, Kurdistan	Child and adolescent welfare agencies	Country of origin sole significant predictor of symptoms improvement in post-traumatic stress. Refusal of asylum was closely associated with higher levels of psychological distress.	Small sample of female UM. Questionnaires used not validated with this population. High percentage loss of data at post-intervention.
Rondung et al., 2022	Sweden	CCT	TF-CBT Group 7 sessions UM 2 sessions caregivers 120 min	*n* = 15 (all UM) Age = 16–20 Mixed (13 male, 2 female) Afghanistan, Eritrea	Multiple sites community setting	Possible decrease in symptoms and increase in well-being from pre-to post- and follow-up assessments.	Small sample size. Very low randomization rate. Loss of follow-up data.
Sarkadi et al., 2017	Sweden	Cohort	TRT (TF-CBT based) Group 4 weeks 5 sessions UM 2 sessions caregivers 90–120 min	*n* = 46 (all UM) Age = 14–18 Mixed (43 male, 3 female) Afghanistan, Syria	Community setting	Significant reduction in PTSD and depression symptoms.	No statistical comparison with control or active comparator. Small sample of female UM. No follow-up assessment.
Schapiro et al., 2022	USA	Qualitative	CBT Group 10 weeks	*n* = 16 (all UM) Age = 14–19 Mixed (13 male, 3 female) El Salvador, Guatemala, Honduras	School setting	Central American minors were reluctant to disclose their stories in the group, fearing for family members and their safety, but appreciated group support, learning coping skills, feeling less isolated.	A formal interview may not be the best modality. Small number of female UM.
Solhaug et al., 2023	Norway	Cohort	TRT (TF-CBT based) Group 5 sessions UM 60–120 min	*n* = 147 Mean age = 16.61 Mixed (130 male, 17 female) Afghanistan, Eritrea, Syria	Asylum centre	Life satisfaction significantly increased pre-to-post but not for those whose asylum application had been rejected or who were still awaiting a decision.	Small sample of female UM. No control group.
Unterhetzinberger et al., 2015	Germany	Case series	TF-CBT Individual 12–28 sessions UM 3–23 sessions caregivers 50–100 min	*n* = 6 Age = 16–18 Mixed (4 male, 2 female) Somalia, Afghanistan, Iraq	Outpatient clinics	Significant reduction in PTSD symptoms	Small sample size. UM not recruited randomly. No follow-up assessment. No statistical comparison with control or active comparator.
Unterhetzinberger & Rosner, 2016	Germany	Case study	TF-CBT Individual 12 sessions	*n* = 1 Age = 17 Female East Africa	Outpatient setting	Non-significant reduction PTSD, depression and anxiety symptoms maintained at 6 months.	Results not generalizable because of single case study
Unterhetzinberger et al., 2019	Germany	Cohort	TF-CBT Individual 15 sessions UM 8 sessions caregivers 100 min	*n* = 26 (all UM) Age = 15–19 Male Afghanistan, Eritrea, Gambia, Iran, Sierra Leone, Somalia, Sudan, Syria	Outpatient clinic	Significant reduction in PTSD and depression symptoms	Small sample size. No statistical comparison with control or active comparator. No female UM.
Van Es et al., 2021	Netherlands	Cohort	Multimodal Individual 8 sessions (average) 80 min	*n* = 41 (all UM) Age = 12–19 Mixed (27 male, 14 female) Eritrea, Syria, Afghanistan	Living location of UM or location of choice	Decrease in PTSD symptoms. Professionals evaluated the treatment positively.	Small sample size. Low treatment adherence.
Van Es et al., 2023	Netherlands	Multiple baseline case series	Multimodal Individual 8 sessions 80 min	*n* = 10 (all UM) Age = 15–18 Mixed (8 male, 2 female) Eritrea, Syria	Living location of UM or location of choice	No clinically reliable symptom reductions at post-test or follow-up.	Small sample size. Substantial number of dropouts and missing data. Questionnaires not validated.
Van der Gucht et al., 2019	Belgium	Cohort	Mindfulness Based Intervention (MBI) Group 8 weeks 90 min	*n* = 9 (all UM) Age = 13–18 Mixed (4 male, 5 female) Afghanistan, Albania, Somalia	Shelter	MBI may reduce negative affect and improve positive affect and reduce symptoms of depression.	Small sample size. No follow-up assessment. No statistical comparison with control or active comparator.
Vickers, 2005	United Kingdom	Case study	CBT Individual 16 sessions	*n* = 1 Age = 14 Female Africa	Child and adolescent MH service	From severe PTSD to minor symptoms (non-significant reduction)	Results not generalizable. No follow-up assessment.

### Quality assessment

An overview of the quality assessment can be seen in Table 2. The assessment of the quantitative studies (*n* = 17, 94.4%) with the EPHPP tool resulted studies attaining the following scores: “weak” score (*n* = 10, 58.8%), followed by “moderate” (*n* = 5, 29.4%), and strong (*n* = 2, 11.8%). Weak studies scored poorly for blinding, controlling for confounders and selection bias. The assessment of the qualitative component of included studies (*n* = 5, 31.6%) using the CASP tool resulted in studies attaining the following scores: “weak” score (*n* = 1, 20%), “moderate” score (*n* = 2, 40%), “strong” score (*n* = 2, 40%). Weak and moderate qualitative studies did not clarify the theoretical underpinnings (e.g., ontological and epistemological assumptions) of the study; did not clearly explain how data was collected; did not adequately consider the relationship between researcher and participants; did not provide an in-depth description of the analysis to justify that the data analysis is sufficiently rigorous; did not present a clear statement of findings with evidence both for and against the researchers’ arguments; and finally the researchers did not demonstrate how they examined the credibility of their findings (e.g., triangulation, respondent validation, multiple analysts). A more detailed depiction of the EPHPP and CASP appraisals can be found in Online Resource 3 and Online Resource 4.

**Table 2:** Study quality appraisal

Study	Study Design	Quantitative	Qualitative	Mixed Methods	Total Score EPHPP	Total Score CASP
Ehntholt et al. (2005)	Case-control	x			Weak	
King & Said (2019)	Cohort			x	Weak	Weak
Mongelli (2019)	RCT	x			Moderate	
Patel et al. (2022)	Cohort	x			Weak	
Pfeiffer & Goldbeck (2017)	Cohort	x			Weak	
Pfeiffer et al. (2018)	RCT	x			Strong	
Pfeiffer et al. (2019)	RCT	x			Strong	
Rondung et al. (2022)	CCT	x			Weak	
Sarkadi et al. (2017)	Cohort			x	Moderate	Strong
Schapiro et al. (2022)	Qual		x			Strong
Solhaug et al. (2023)	Cohort	x			Moderate	
Unterhitzenberger et al. (2015)	Case series	x			Weak	
Unterhitzenberger & Rosner (2016)	Case study	x			Weak	
Unterhitzenberger et al. (2019)	Cohort	x			Moderate	
Van Es et al. (2021)	Cohort	x			Weak	
Van Es et al. (2023)	Multiple Baseline Case Series			x	Weak	Moderate
Van der Gucht et al. (2019)	Cohort			x	Moderate	Moderate
Vickers (2005)	Case study	x			Weak	

### Quantitative results

#### Improvement in QoL

Of the 18 studies, only two (11.1%) used quantitative measures for assessing QoL [60, 61]. Both studies used the Cantril Ladder [62] to assess life satisfaction. Authors reported that TF-CBT seems to significantly increase life satisfaction, but only for UM whose asylum application had not been rejected or who were still awaiting a decision [61]. In another study, authors did not undertake statistical testing as the scope of their study was feasibility rather than effectiveness. Nonetheless, the authors reported improvement in mean scores on the Cantril Ladder following TRT, at post-intervention and at 18-weeks follow up [60].

#### Improvement in psychopathology symptoms

##### Cognitive behavior therapy (CBT)

Three studies (16.7%) examined CBT in UM [63–65]. Group CBT appeared to result in a significant decrease in overall PTSD symptoms severity and intrusive PTSD symptoms. UM also appeared to improve with regards to behavior and emotional difficulties [63]. Unfortunately, these improvements were not maintained at 2-month follow-up [63]. In a case study, the author reported that after sixteen sessions of individual CBT, the UM had moved from a severe PTSD score to minor symptoms although this was not considered a clinically significant reduction [64].

##### Trauma-focused cognitive behavior therapy (TF-CBT)

Ten studies (55.6%) examined TF-CBT in UM. TF-CBT appeared to reduce PTSD symptoms in most studies (*n* = 7) [66–72] with medium to large effect sizes [68–70] and improvements were maintained at two weeks to 6-months follow-up [61, 67, 68, 73]. Improvements were mostly observed in the domains of re-experiencing and avoidance, as well as alterations in cognitions and mood [69]. TF-CBT also appeared to reduce depression symptoms with medium [70] to large effect sizes and maintained at 6-months-follow-up [67, 68, 71]. Reductions in anxiety symptoms [67] and improvements in emotional difficulties [72] were also noted.

##### Third wave cognitive behavioral interventions

Three studies (16.7%) were classified as third wave CBT interventions [74–76]. A third wave group intervention (i.e., CBT, ACT, DBT, CFT) with UM (*n* = 14), seemed to observe significant improvement in behavioral difficulties but not in PTSD symptoms [74]. Researchers conducting an MBI group intervention (*n* = 9), appeared to significantly reduce negative affect of UM with a medium effect size [75]. An ACT group intervention with UM (*n* = 12), seemed to result in significant reduction in PTSD and depression symptoms in the intervention group compared to the control group (no intervention) and authors noted increases in psychological flexibility and mindfulness with very large effect size [76].

##### Multimodal approaches

Two studies (11.1%) reported on programs that provided a range of strategies to improve MH in UM [77, 78]. A multimodal trauma-focused treatment approach, (*n* = 41) appeared to result in significant decreases in PTSD symptoms with a very large effect size [77]. An overview of the reported outcomes of included studies can be found in Online Resource 5.

### Qualitative results

Of the 18 studies included, only five (27.8%) investigated treatment acceptability of the intervention. Characteristics of the included studies can be found in Online Resource 6. The QRS resulted in the development of two core categories (see Table 3). The category “Treatment components” contains three themes and describes various aspects of the treatment such as tools and coping strategies used by participants, the adaptability of the treatments and how acceptable the interventions were perceived to be. The “Treatment outcomes” category contains two themes and reflects what participants reported gaining from the interventions. Outcomes included an abatement of emotional symptoms and additional benefits observed by participants such as the sense of belonging. These themes are situated within an overarching concept “Creating safety and cultivating togetherness”. The distribution of themes across included papers are presented in Table 4. All quotations are taken from the study participants, therapists, or stakeholders, unless specified as an author comment.

**Table 3:** Categories, themes, and overarching concept

Category	Theme	Overarching Concept
Treatment components	Tools/Strategies Adaptability Acceptability	Creating safety and cultivating togetherness
Treatment outcomes	Emotional symptoms Additional benefits	

**Table 4:** Distribution of themes across qualitative studies

Author and date	Treatment components			Treatment outcomes	
	Tools/ Strategies	Adaptability	Acceptability	Emotional symptoms	Additional benefits
Sarkadi et al. (2017)	√			√	√
King & Said (2019)	√			√	√
Van der Gucht et al. (2019)	√	√	√	√	
Schapiro et al. (2022)	√	√	√	√	√
Van Es et al. (2023)		√	√	√	√

#### Treatment components

This category includes three themes: the tools and strategies that participants found useful, adaptability of therapists and interventions used, and how acceptable participants perceived the interventions to be.

##### Tools and strategies

This theme depicts feedback of UM on the tools and strategies learned from CBT and third wave interventions. The exercises were considered valuable, and UM incorporated them into their daily lives. In four studies, UM assimilated new tools learned with previously established helpful strategies, whereas other UM seemed to replace maladaptive coping mechanisms with more adaptive methods.

“These exercises help me a lot to handle my fear and anxiety. I get these attacks every night and I think if I had not done these exercises I would have been even worse.” [71].

“When I can’t concentrate at school or when I go to sleep at night, I do an exercise. It helps.” [75].

“The mindfulness exercises are helpful when I can’t concentrate with school or when I have a lot of stress. I do some exercises. At night I prefer to pray.” [75].

“After doing exercises I’ve learned here, I’ve reduced sleeping pills.” [71].

Although four studies reported that some UM found the tools learned indispensable, it was also noticed by three studies that it is not always easy to change already established coping mechanisms.

“To practice the exercises was quite difficult to do because I barely did it on my own so. It was just something that didn’t cross my mind. I didn’t think about practicing.” [75].

In one study authors reported that “other useful strategies mentioned were distraction, such as listening to music, or activation, such as hanging out with friends or talking to someone they trusted.” In other cases however, “one of the young people stated that nothing can be done about the situation except to endure” [71]. Furthermore, a therapist in another study reported that “despite lively discussion about the trauma narrative, in the end, they mostly felt, it’s still probably good to avoid things” [65].

##### Adaptability

This theme illustrates the ways in which therapists adapted the intervention protocols to meet the needs of UM. Clinicians in a study reported that they would improvise to engage young people and use exercises from other training they had attended or that “had been shared informally by colleagues”. Some therapists felt it “was not productive to push them to talk about their worst experiences if they were not ready”. For instance, facilitators would emphasize “coping skills and address acculturation stress over disclosure of trauma”. Furthermore, one therapist “found herself moving away from CBT and using an intervention from her own culture” [65].

“Like the skills, you really have to twist how you present or how you teach them because for me… it has been more effective to sit with a group of students from Guatemala and burn sage than talk about the CBT triangle.” [65].

Furthermore, adaptability also concerned the measures that the research team took to engage participants. For instance, in another study reported that UM “appreciated the outreach work as five participants stated they would not have participated if they had to travel to a mental health institution” [78]. The dedication shown from clinicians was an important factor in participant engagement.

“They come back, and again, they don’t give up. Sometimes I was tired and wanted to sleep and did not want to talk, but they came back and helped me and little by little, I started talking.” [78].

Another adaptation when working with this population may be individual attention to some UM in addition to group treatments. Authors reported that some UM “would have liked more individual conversations with the group facilitators” [71]. Other authors observed that “one youth stated he preferred to talk about some issues alone with the therapist” [65].

##### Acceptability

Findings related to acceptability were mixed throughout three studies. Whilst in one study authors reported that “most participants found the treatment helpful and would recommend it to others” [78], clinicians in another study expressed that the intervention “didn’t quite land” for the young people [65].

“It is boring. If something is not interesting you don’t want to stay, you want to go. For me it was difficult to concentrate and nothing changed between the first and second session.” [75].

“I went to one session, I see the exercise and it was kind of interesting. We had to think and this was good. It was not difficult but I don’t need it. This is just not for me.” [78].

Furthermore, authors reported that in their study “participants mentioned how the language of the MBI program has hampered an adequate participation” [75]. Nonetheless, authors mentioned that “both participants mention how this language barrier seemed to be slightly reduced by the possibility to interact with the trainer. Furthermore, this interaction made it possible for the participants to obtain additional information about the concepts and attitudes of the practice of mindfulness, which they experienced as helpful.”

#### Treatment outcomes

This category includes two themes: the decrease of internal private events (i.e., emotions, thoughts), and additional benefits that UM observed from the interventions.

##### Emotional symptoms

The abatement of emotional symptoms is a theme that was apparent throughout all included studies. Participants expressed noticing a decrease in anxiety, stress, and sadness. UM also mentioned an improvement in concentration and a feeling of calmness. Moreover, researchers noted that participants “reported having been helped with sleeping difficulties, intrusive memories, depressed thoughts, fear, and irritation” [71].

‘Before I came to the group I felt very sad and worried, since coming here I have opened up and I feel relaxed’ [74].

“The calmness you felt after doing these exercises, it was great to be able to feel this calm.” [71].

“For me the most important thing was that when you are down or you are in a mental crisis, how to get yourself out of it, how to save yourself somehow.” [71].

“Before I wasn’t interested, I didn’t feel like doing anything. I didn’t want to go to school. Or didn’t go to appointments. Sleeping was also difficult. After and during the treatment, I feel like I’m more keen, I go to school and to the appointments.” [78].

Throughout the studies however, there was also an overwhelming need for UM to learn how to control their internal private events.

“When I came to this group I had no control of myself, because everything I had been through affected me and was controlling me. The group helped me to gain control over myself.” [71].

“I can control stress and I can control my fear, I can control that I cannot sleep, but I have difculty controlling my anger. That’s really difficult to control.” [71].

##### Additional benefits

This theme included other benefits that participants felt they had gained from the treatments. Authors in one study reported that participants could “address difficult topics with loves ones and experienced an improved relationship with friends and family. Other benefits included feeling proud of themselves and improved self-care” [78].

“I learn to appreciate things, and they taught me how to give advice to other people.” [65].

“Now I have a chance to see all different parts of my life, the good and the bad. This offers me balance.” [78].

Moreover, some UM also conveyed a feeling of having developed a new identity following the treatment. Authors explained that UM were able to assimilate their previous experiences into their present identity, by discussing issues with peers and considering themselves in relation to the culture of the host country. Importantly, UM also experienced a sense of belonging following the treatment they received [71].

“From the first time I came to Sweden, I feel that I am a newborn, now I belong to this world as well.” [71].

However, despite improvements in emotional symptoms and other benefits, authors reported that “many of the boys expressed dejection and described how incidents in the home country affected them” [71]. The impact of current stressors was also evident and had a negative impact on treatment benefits. Authors reported that most UM “spoke about the daily stressors they continued to experience, including worries about the future, anxiety concerning family reunification, troubles with peers, and worries about the lives and wellbeing of family members.” [78].

#### Overarching concept: creating safety and cultivating togetherness

The two core categories are nested within an overarching concept of creating safety and cultivating togetherness. UM expressed feeling less alone through the process of sharing life narratives. UM felt support by peers and therapists, and they reported that the groups created a sense of safety. Authors reported that UM “described a trust in the group and the fact that it felt safe to open up” [71]. In another study authors also reported that UM “explained they felt free to discuss their experiences, were relieved after talking, and felt space to discuss subjects they would not address with others” [78].

“I come here and saw everyone that has problems just like me. Everyone is working together to help each other.” [74].

“We were so comfortable in the group, we could talk to each other like a friend, like a brother. It was very nice in this group, and it was right …” [71].

“You thought you were alone with these thoughts but then when you got into the group and saw the others, you felt, yes, but now it feels a bit easier because I’m not alone with this problem.” [71].

“For me, it was good because they shared things about their lives, and you can learn from another person. I thought I was all alone, but there are other people who are alone too, without their parents, but with the help of another person sometimes, we are able to keep going.” [65].

“Since I started the groups, I feel my nightmares don’t get to me as often. I feel a bit safer.” [65].

The importance of feeling safe and less alone was very apparent in a study where this was lacking as UM reported not feeling supported, in comparison to other studies.

“We were only two persons. Sometimes I was alone. Normally I like to be in a group. When I was alone, I was just, I was not able to think anymore.” [75].

## Discussion

A total of 18 studies were included in this review examining the identified evidence on the effectiveness and acceptability of CBT and third wave interventions at improving the QoL and psychological well-being of UM. The first objective was to explore whether CBT interventions improve QoL in UM. From the studies included, only two utilized quantitative measures of QoL [60, 61]. Both studies in this review utilized the Cantril Ladder to examine well-being. Despite the Cantril Ladder being one of the most widely administered measures to assess well-being, the scale tends to focus on power and wealth rather than more general well-being [79]. Thus, it should not be considered appropriate for use with UM and more adolescent friendly measures should be adopted by future studies. In addition, the emphasis on symptom reduction and diagnosis rather than QoL, was also evident by the fact that many studies required participants to have either PTSD symptoms (*n* = 10) or PTSD diagnosis (*n* = 4) to be included. Although it has been suggested to limit trials to one diagnosis to reduce skewing results [80], this can be problematic for several reasons. Excluding individuals who do not have a diagnosis prevents UM from receiving interventions that can improve their overall well-being. Moreover, UM often under-report symptoms or attempt to suppress any expressions of negative emotions due to fears that this might compromise their asylum application [81]. Thus, requiring diagnosis for inclusion in research, may inadvertently exclude certain UM from receiving support. Regardless of whether UM fulfil a diagnosis or not, it is imperative that their difficulties are addressed to improve functioning and general well-being [82].

The second objective was to explore acceptability of CBT and third wave interventions. Only five studies investigated acceptability with participants [65, 71, 74, 75, 78] using qualitative methodology. Similar to previous reviews [80], results indicate that even in studies where no significant quantitative symptom improvements were observed, the qualitative data still provided evidence for progress in other domains (i.e., improved interpersonal relationships and self-care; [78]). In contrast to previous studies [45], this was the first review to use QRS to go beyond simple aggregation of findings. The QRS results demonstrated that CBT and third wave interventions are both acceptable to UM and able to improve emotional symptoms. However, beyond improving emotional symptoms and providing UM with additional benefits through valuable coping strategies, it appears that what these interventions had in common is the ability to create a safe space for UM and cultivated a sense of togetherness. We argue that these feelings of safety and togetherness stemming from CBT interventions, are in fact the precursor and catalyst for change which ultimately lead to improved well-being and QoL in UM. Unfortunately, results should be interpreted with caution as not all qualitative components of studies included were of high quality. Recommendations to researchers carrying out mixed-methods or qualitative studies in UM would be to follow the CASP tool [59] as a checklist to provide higher quality research and reporting.

The final objective was to examine the identified evidence on effectiveness of interventions in improving psychological well-being in UM. Overall, TF-CBT appears to have the most evidence pointing to effectiveness in the enhancement of psychological well-being of UM, either as an individual or group modality [67–70, 72, 73]. It is unclear whether TF-CBT is superior to traditional CBT or other third wave approaches as it has yet to be directly compared to another treatment approach. Significant improvements in MH and psychological flexibility were also observed for third wave interventions [74, 76]. These results are in line with recommendations to adopt transdiagnostic approaches for UM [83].

Despite encouraging findings, the quantitative studies had several methodological drawbacks. Similar to previous reviews [84], the significance and generalizability of the results is limited by small sample sizes. Additionally, most studies were either cohort studies, case series, or case reports and thus it is difficult to evaluate the effectiveness of the interventions if they are uncontrolled [84]. Previous studies have advocated for single-blinded controlled trials as a possible solution for this population [73, 84]. In addition, only seven studies (38.9%) included follow-up assessments in their study designs limiting the evidence regarding long-term benefits of the interventions [60, 61, 63, 67, 68, 73, 78]. Improvements from the interventions were only maintained for four studies (22.2%) [61, 67, 68, 73]. Previous studies have suggested a possible time-lapse between symptom reduction and functional improvement [85] but this requires further exploration considering the lack of follow-up assessments in current literature. Moreover, most studies included were carried out in community or outpatient settings. Since a structural barrier to treatment for UM is poor access to services, future research should prioritize offering services in locations where UM are residing [75–78]. This was found to be an important part of acceptability as UM reported that they “appreciated outreach work and stated they would not have participated if they had to travel to a mental health institution” [78]. Other areas of concern included: lack of female participants, the exclusion of UM with severe mental illness (SMI), and UM who do not speak the language of the host country. Female UM have a higher risk of being sexually exploited and abused when they flee their homes [86], and thus female gender may be a risk factor for the development of PTSD and depression [4]. Despite this knowledge, female UM are still extremely underrepresented in the current literature and gender differences in UM are under-researched [87]. Future studies should examine differences between male and female UM to determine whether gender-sensitive interventions should be prioritized. Another concern was that the most common exclusion factor was UM with SMI (i.e., psychosis or acute suicidality; [45]). Arguably, this excludes some of the most vulnerable UM, and those who may benefit the most from a psychotherapeutic intervention. Additionally, excluding UM who do not speak the language of the host country, also prevents a great number of UM from receiving support [63, 69, 70, 73, 75]. Studies that used interpreters, cultural mediators, or used bilingual therapists reported significant decreases in PTSD and depression symptoms in UM who did not speak the language of the host country, suggesting that UM should not be rejected due to language where possible [45].

As the methodological drawbacks observed in this study have been reported in previous literature between 2018 and 2021 [45, 80, 84, 87] it is alarming that significant strides towards addressing these limitations have yet to be addressed. The limited evidence on the long-term impact of interventions for UM is also profoundly concerning. In view of the atrocities currently occuring across the globe, this study calls for a drastic increase in high quality research in this field. However, the prominent difference between quantitative and qualitative results indicates that perhaps RCTs and “controlled” studies should no longer be considered the ‘gold standard’ when evaluating interventions with this population and that a drastic shift in methodology is required. In contrast to RCTs advocating for a “one-size-fits-all” approach, we suggest that researchers should strive for a deeper, more personal understanding of how to enhance well-being and functionality of UM. Based on the existing evidence-base of third wave interventions and initial but promising outcomes with UM, we suggest implementing therapeutic approaches that can be tailored to each individual. Third wave interventions achieve this by exploring the function of problematic behavior whilst taking context into account, with the aim to alleviate suffering and encourage valued living [36]. Furthermore, examining processes of change (i.e., session by session) and applying within-person longitudinal methods (i.e., six months follow-up) can elucidate how individual treatment components affect specific UM in their unique contexts. Such advancements in methodology can provide high quality data whilst simultaneously providing clinicians with comprehensive insight regarding the needs of UM to ultimately improve their quality of life.

### Limitations

The high risk of bias in some of the included studies should be considered when interpreting the findings of this review. Furthermore, although languages other than English (i.e., Greek, Italian, French) were incorporated in the inclusion criteria of this systematic review, several potentially relevant other-language articles were excluded (e.g., some German-language studies).

## Conclusion

This review is the first to examine the evidence on effectiveness and acceptability of CBT and third wave interventions at improving the QoL and psychological well-being of UM. TF-CBT appeared to have the most evidence demonstrating effectiveness in ameliorating symptoms of PTSD, depression, and anxiety. Outcomes from third wave CBT interventions are encouraging but further research is required to establish effectiveness. CBT and third wave interventions create safety and cultivate togetherness which ultimately leads to increased well-being and QoL in UM. This review highlights the lack of examining QoL and acceptability of interventions in UM. Importantly, there is a great need for more methodologically robust quantitative and qualitative studies. It is also suggested that future researchers should utilize mixed methods methodologies to examine effectiveness and acceptability of interventions. Finally, we recommend that future studies should aim to include more females in their sample to examine gender differences to provide higher quality psychological support for this underserved population.

## Electronic supplementary material

Below is the link to the electronic supplementary material.

Supplementary Material 1

## Data Availability

The data supporting the findings of this study are available in Open Science Framework (10.17605/OSF.IO/Y3QVM).
