# Supplementary material for: Examining cognitive behavioral therapy interventions for unaccompanied minors: a systematic review and qualitative research synthesis
Source: Eur Child Adolesc Psychiatry. 2024 Jun 27;34(2):465–81. doi: 10.1007/s00787-024-02500-z (PMC11868330; doi:10.1007/s00787-024-02500-z)
Supplement: Supplementary file 1 — Supplementary Material 1 [file 787_2024_2500_MOESM1_ESM.docx]

**Examining Cognitive Behavioral Therapy Interventions for Unaccompanied Minors: A Systematic Review and Qualitative Research Synthesis**

Dafne Morroni; Pinelopi Konstantinou; Chrysilia Gkleka; Angelos P. Kassianos, and Maria Karekla

Correspondence concerning this article should be addressed to Maria Karekla, Ph.D., Department of Psychology, University of Cyprus, PO Box 20537, 1678, Nicosia, Cyprus. Email: [mkarekla@ucy.ac.cy](mailto:mkarekla@ucy.ac.cy)

**Supplementary Materials**

**Online Resource 1.** Search Strategy

| Database | Search Terms Used |
| --- | --- |
| Pubmed | (("refugee"[Title/Abstract]) AND ("child"[Title/Abstract])) AND ("therapy"[Title/Abstract]) |
| Scopus | refugee  OR  asylum  AND seeker  AND  cognitive  AND behavior  AND therapyAND  minors |
| Embase | ('refugee'/exp OR refugee) AND ('child'/exp OR child) AND ('cognitive behavioral therapy'/exp OR 'cognitive behavioral therapy') |
| PsycInfo & PsycArticles & Open Dissertations | refugees or asylum seekers or displaced or migrants or immigrants or emigration AND children or adolescents or youth or child or teenager AND therapy or treatment or intervention or counseling or psychotherapy AND cognitive behavioral therapy or cbt or cognitive behavioural therapy |
| ProQuest Psychology Database | ab(Refugee) AND ab(Unaccompanied Minor) OR ab(unaccompanied youth) AND ab(cognitive and behavioral and therapy) AND ab(third wave) |

**Online Resource 2.** Data Extracted

Data extracted included: (1) Author(s), year of publication, study location; (2) residence status (refugee, asylum-seeker, unaccompanied); (3) study design (case-control, cohort, randomized controlled trial, controlled clinical trial, case series, case study); (4) group assignment (randomized, matched, convenience, no control group); (5) age range; (6) percentage of female participants; (7) psychopathological difficulties (PTSD, depression, anxiety, complicated grief, with/without diagnosis); (8) exclusion of severe cases and reasons for exclusion (suicidal, severe symptomatology, psychosis, language, other); (9) type of intervention (CBT/TF-CBT/Third wave); (10) Modality (Individual or group therapy); (11) dose of intervention (in 50 minute sessions); (12) number and duration of follow-up assessments; (13) control condition (waitlist/no treatment, unspecific treatment like support or counselling, active vs. inactive control); (14) outcome measures (any quantitative assessment for measuring changes in psychopathological difficulties); (15) Effect sizes; (16) improvements reported by authors, (17) interpreters used; (18) limitations.

**Online Resource 3.**  Risk of Bias of included studies based on EPHPP

*Note.* √ = Strong, / = Moderate, X = Weak, n/a = Not applicable. Strong = No weak ratings, Moderate = one weak rating, Weak = two or more weak ratings.

| Studies | Selection Bias | Study Design | Confounders | Blinding | Data Collection | Withdrawals & Dropouts | TOTAL SCORE |
| --- | --- | --- | --- | --- | --- | --- | --- |
| Ehntholt et al. (2005) | X | / | X | X | √ | √ | WEAK |
| King & Said (2019) | / | / | X | X | √ | √ | WEAK |
| Mongelli (2019) | √ | √ | / | X | √ | √ | MODERATE |
| Patel et al (2022) | / | / | X | X | √ | X | WEAK |
| Pfeiffer & Goldbeck (2017) | √ | / | X | X | √ | √ | WEAK |
| Pfeiffer et al. (2018) | √ | √ | √ | / | √ | √ | STRONG |
| Pfeiffer et al. (2019) | √ | √ | √ | / | √ | √ | STRONG |
| Rondung et al. (2022) | / | / | X | X | √ | √ | WEAK |
| Sarkadi et al. (2017) | / | / | X | X | √ | √ | WEAK |
| Solhaug et al. (2023) | √ | / | √ | X | √ | / | MODERATE |
| Unterhitzenberger et al. (2015) | X | X | X | X | √ | √ | WEAK |
| Unterhitzenberger & Rosner (2016) | X | / | X | / | √ | X | WEAK |
| Unterhitzenberger et al. (2019) | / | / | X | X | √ | √ | WEAK |
| Van Es et al. (2021) | / | / | X | X | √ | √ | WEAK |
| Van Es et al. (2023) | X | / | n/a | X | √ | / | WEAK |
| Van der Gucht et al. (2019) | √ | / | √ | X | √ | √ | MODERATE |
| Vickers (2005) | √ | X | X | X | √ | n/a | WEAK |

**Online Resource 4.** Appraisal of Qualitative Studies using CASP.

| CASP Questions | | | | | | | | | | | | Total CASP |
| --- | --- | --- | --- | --- | --- | --- | --- | --- | --- | --- | --- | --- |
| Studies | 1.Was there a clear statement of the aims of the research? | 2. Is a qualitative methodology appropriate? | 3. Was the research design appropriate to address the aims of the research? | 4. Are the study’s theoretical underpinnings clear, consistent, and conceptually coherent? | 5. Was the recruitment strategy appropriate to the aims of the research? | 6. Was the data collected in a way that addressed the research issue? | 7. Has the relationship between researcher and participants been adequately considered? | 8. Have ethical issues been taken into consideration? | 9. Was the data analysis sufficiently rigorous? | 10. Is there a clear statement of findings? | 11. How valuable is the research? |  |
| King & Said (2019) | Somewhat | Yes | No | No | Somewhat | No | No | Somewhat | No | Somewhat | Somewhat | Weak |
| Sarkadi et al. (2017) | Yes | Yes | Yes | Yes | Somewhat | Yes | No | Yes | Yes | Yes | Yes | Strong |
| Schapiro et al (2022) | Yes | Yes | Yes | Yes | Yes | Yes | No | Yes | Somewhat | Somewhat | Yes | Strong |
| Van Es et al (2023) | Yes | Yes | Yes | No | Yes | Yes | No | Yes | Somewhat/No | Somewhat | Yes | Moderate |
| Van der Gucht et al. (2019) | Yes | Yes | Yes | Can’t tell | Somewhat | Somewhat | No | Yes | No | Somewhat | Yes | Moderate |

*Note.* 1-4 ‘Yes’ = Weak, 5-7 ‘Yes’ = Moderate, 8-10 ‘Yes’ = Strong. ‘Can’t tell’ means when there is insufficient information reported to make a judgement (i.e., a reporting issue). ‘Somewhat’ means when relevant information including both strengths and limitations was provided (i.e., a methodological issue).

**Online Resource 5.** Table of outcomes.

| Study | Measures | Pre-intervention Mean (SD) | Post-intervention Mean (SD) | Statistically significant? | Control Pre-intervention Mean (SD) | Control Post-intervention  Mean (SD) | Statistically significant? | Clinically significant? | Follow up |
| --- | --- | --- | --- | --- | --- | --- | --- | --- | --- |
| Ehntholt et al., 2005 | R-IES  DSRS  RCMAS  SDQ | 39.80 (8.40) 12.33 (4.70) 16.87 (7.22)  9.20 (7.76) | 33.80 (9.71) 11.67 (3.62) 14.67 (7.12)  5.40 (4.35) | Yes (p=0.01) No (p=0.10) No (p=0.14) Yes (p<0.05) | 38.55 (8.37) 12.00 (5.37) 16.16 (6.57)  6.43 (4.69) | 42.18 (9.38) 13.00 (6.57) 18.91 (6.04)  5.43 (4.28) | No (p=0.07) No (p<0.05) No (p=0.07) No (p>0.05) | Remained clinical PTSD Remained subclinical anxiety and depression | Not maintained at 2 months |
| King & Said, 2019 | SDQ CRIES-8 | 16.50 (6.28) 30.67 (5.50) | 9.33 (5.50)  23.60 (7.46) | Yes (d=1.08) large effect size  CRIES-8 No |  |  |  | Yes No | None |
| Mongelli, 2019 | I-CAMM  I-AFQ-Y  BDI-II  CAPS-CA | 15.33 (6.25) 17.16 (5.67) 17.50 (7.68) 43.83 (27.11) | 29.00 (3.94)  5.00 (1.67)  6.33 (2.42)  16.33 (11.72) | Yes (p=0.001) very large effect size (d=1.57) Yes (p=0.001) very large effect size (d= 1.62) Yes (p=0.007) Yes (p=0.046) | 27.66 (4.50) 10.16 (3.31)  3.60 (4.98)  32.50 (16.74) | 27.66 (4.96) 11.66 (2.50)  3.33 (3.61)  35.50 (18.11) | No  No  No  No | Significant reduction in PTSD and depression symptoms. Significant increase in psychological flexibility and mindfulness | None |
| Patel et al., 2022 | CATS  SDQ  CATS PM | 12.60 (11.70). 11.30 (6.46)  4.77 (3.74) | 4.60 (4.98).  8.10 (5.80)  3.20 (3.70) | Yes (p <.001) very large effect size (d=-1.03) Yes (p=0.002) medium effect size (d=-.53) Yes (p=0.002) small effect size d=.44 |  |  |  | Yes | None |
| Pfeiffer & Goldbeck, 2017 | CATS | 27.58 (7.88) | 20.67 (6.30) | Yes (p<0.001) large effect size (d=0.97) |  |  |  | Average remained clinical PTSD, 10 children became subclinical | None |
| Pfeiffer et al., 2018 | CATS  PHQ8 | 29.97 (1.22) 11.52 (0.71) | 23.53 (1.77)  8.25 (0.75) | Yes (d=0.61, p=0.003), medium effect size Yes (d=0.63, p=0.002), medium effect size | 31.85 (1.23) 11.47 (0.71) | 30.27 (1.73) 11.76 (0.76) | No  No | No | None |
| Pfeiffer et al., 2019 | CATS  PHQ8  CPTCI-S | 29.91 (1.16) 11.52 (0.71) 13.18 (0.91) | 23.44 (1.81)  8.28 (0.77)  9.06 (1.06) | Yes (p<0.001, d=0.62) medium effect size Yes (p=0.001, d= 0.62) medium effect size Yes (p<0.001, d=0.59) medium effect size |  |  |  | Country of origin significant predictor of the change in PTSS Baseline levels of depression significant predictor of change in depression | Improvements were stable at 3 months |
| Rondung et al., 2022 | CRIES-13 PHQ-9 GAD-7 GSE  Cantril Ladder | 31.71 (12.06) 12.29 (5.61)  8.57 (5.36)  26.64 (6.63)  5.07 (3.05) | 28.57 (15.67) 7.43 (5.25)  5.14 (5.11)  26.14 (7.38)  7.43 (2.99) | Unavailable data |  |  |  | Possible decrease in symptoms and increase in well-being from pre to post and follow up assessment | 18 weeks |
| Sarkadi et al., 2017 | CRIES MADRS-S | 29.02 (6.33) 29.26 (10.34) | 25.93 (5.96) 23.39 (10.55) | Yes (p=0.017) Yes (p=0.001) |  |  |  | No | None |
| Solhaug et al., 2023 | Cantril Ladder | 4.34 (2.79) | 4.77 (2.52) | Yes (p<0.01) |  |  |  |  | 5.12 (2.76) p<0.01 |
| Unterhitzenberger et al.,2015 | CAPS-CA  PDS | 52  32 | 14.5  12.0 | Yes (p<0.001) |  |  |  | PTSD reduced to subclinical | None |
| Unterhitzenberger & Rosner, 2016 | CAPS-CA  UCLA PTSD  CDI  SCARED | 50  37  21  14 | 5  6  16  17 | Only 1 participant |  |  |  | PTSD, depression and anxiety reduced to subclinical | Improvement maintained at 6 months |
| Unterhitzenberger et al.,2019 | CATS MFQ | 30.58 (7.16) 13.32 (4.26) | 20.16 (11.63) 5.63 (4.52) | Yes (p=0.03) large effect size (d=1.08) Yes (p<0.001) large effect size (d=1.75) |  |  |  | Significant reduction in PTSD and depression symptoms | Yes - immediate 6 weeks and 6 months |
| Van Es et al., 2021 | PHQ-A CRIES-13 | 10.35 (7.17) 42.59 (12.13) | 8.82 (8.49) 19.93 (19.13) | No (p=0.51) Yes (p<0.001, d=1.32) very large effect size |  |  |  | Decrease in PTSD symptoms | None |
| Van Es et al., 2023 | CRIES-13 PHQ-A | 23.2 (20.6) 7.1 (6.4) | 17.3 (17.6). 8.2 (6.6) | No |  |  |  |  | 19.9 (26.2) 10.1 (9.2) |
| Van der Gucht et al., 2019 | I-PANAS-SF Negative Positive DASS-21-D  CRIES | 16.00 (6.50) 15.00 (4.24) 10.89 (4.78) 22.38 (12.54) | 11.56 (3.00) 18.00 (3.84)  6.89 (4.17)  21.88 (5.62) | Yes (p=0.04, g=0.79) medium effect size  No (p=0.07, g=0.71) medium effect size  No (p=0.07, g=0.81) large effect size No |  |  |  | No- Preliminary results that MBI may reduce negative affect and improve positive affect and reduce symptoms of depression | None |
| Vickers, 2005 | PDS | 42 | 9 | Only 1 participant |  |  |  | PTSD scores reduced to subclinical | None |

**Online Resource 6.** Comparison of qualitative studies.

|  | Sarkadi (2017) | King & Said (2019) | Van der Gucht (2019) | Van Es (2023) | Schapiro (2022) |
| --- | --- | --- | --- | --- | --- |
| Sample | 22 UM | 7 UM | 4 UM | 7 UM | 16 UM & 10 stakeholders |
| Setting | Community setting Sweden | UK mental health service | Refugee shelter Belgium | Specialized MH care institute Germany | Health Center San Francisco |
| Purpose | Acceptability | Acceptability | Experience of MBI | Treatment satisfaction | Safety and acceptability |
| Methods | Narrative inquiry | Narrative inquiry | Narrative inquiry | Narrative inquiry | Narrative inquiry |
| Data collection | Focus group interviews | Semi-structured interviews | Semi-structured interviews | Semi-structured interviews | Semi-structured interviews |
| Analysis | Content Analysis | Thematic Analysis/Unclear | Thematic Analysis | General Inductive Approach | Thematic Analysis |
| Main themes | Six themes: social support, normalisation, valuable tools, comprehensibility, manageability, and meaningfulness | Felt welcome and respected, valued being part of a group of young people in a similar position, learned a lot from the group and felt less stressed since joining | Three themes: expectations of MBI programme, experiences of helping and non-helping factors, mindfulness as coping strategy | Most UM found it useful and positively impacted well-being. Better sleep and concentration. Elevated self-care, feeling proud and engaging in conversations with those close to them | Five themes: Self-protective silence and denial of symptoms, Personal risks of disclosure, marginalizing language, adapting curriculum and delivery, benefits of group |
